# Supplementary material for: Phase precession of spindle-slow oscillation coupling across the human brain
Source: bioRxiv. 2025 Nov 21:2025.11.20.689541. Preprint. [Version 1] doi: 10.1101/2025.11.20.689541 (PMC12667803; doi:10.1101/2025.11.20.689541)
Supplement: Supplement 1 [file media-1.pdf]

SUPPLEMENTARY INFORMATION

eTable 1. Characteristics of Participants

	EMU	AMB	p-value
**N**	58	43	
**Age**			
**mean**	45	73	<0.001
**SD**	16.2	9.1	
**range**	20-80	51-85	
**Sex (% Male)**	26	44	0.08

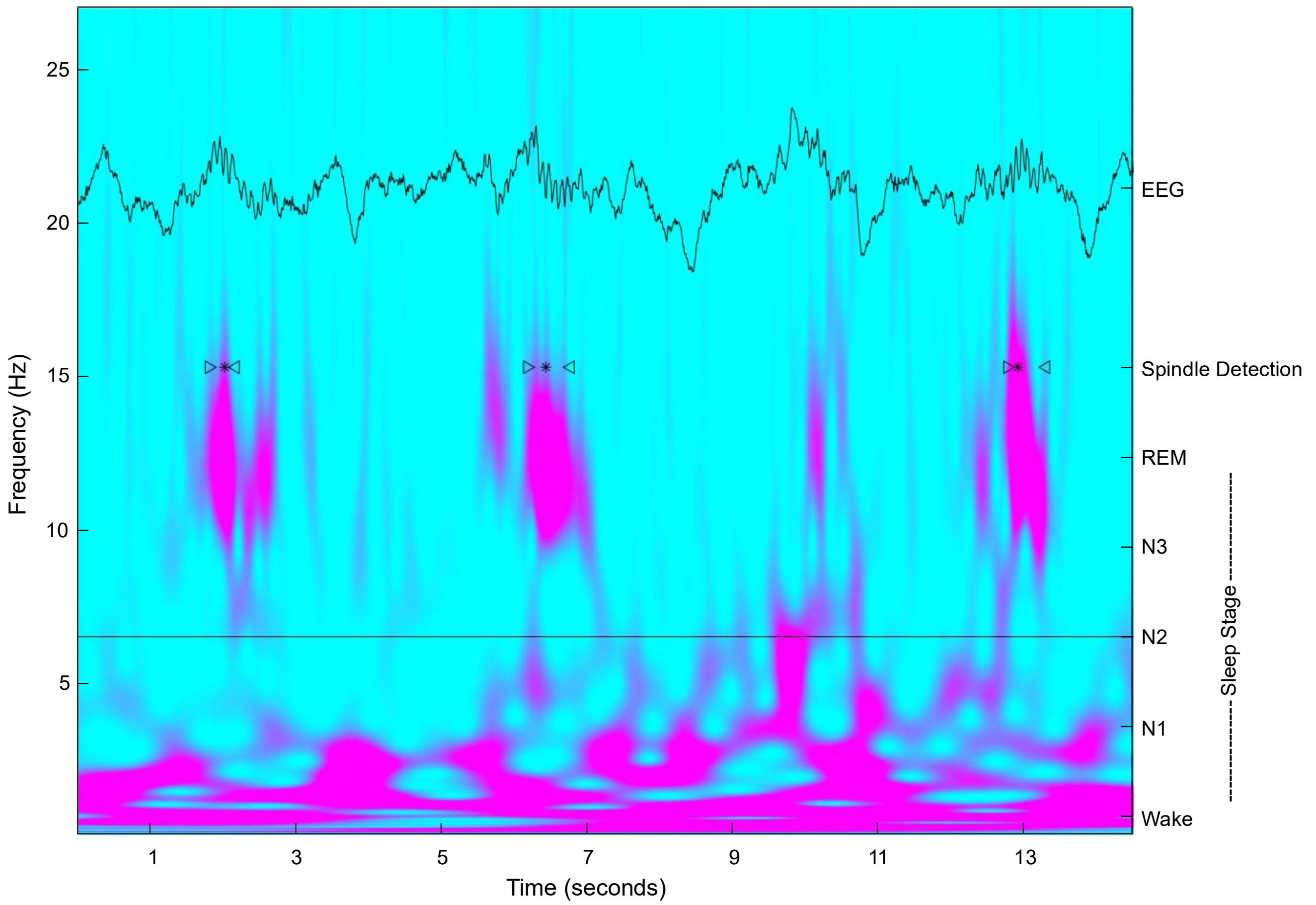

eFigure 1. Spectral analysis of spindle and slow oscillations. A time-frequency spectrogram is shown for 15 seconds of EEG in N2 sleep. The corresponding raw EEG tracing is superimposed (top). Automatic spindle detections are indicated by the >*< symbols. In this epoch, 3 automatically-detected spindles are seen, each occurring near the peak of the SO signal.

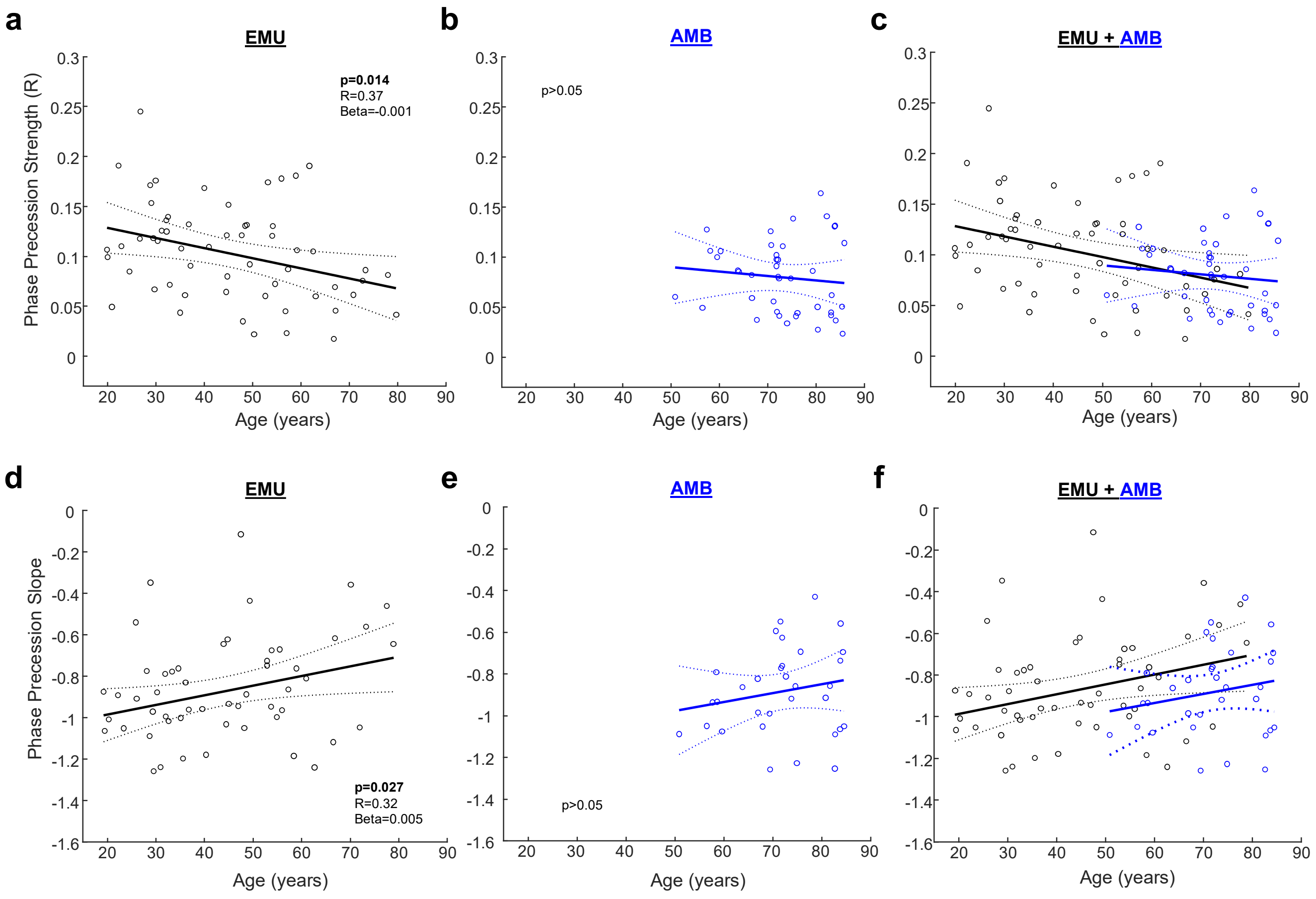

eFigure 2. Sub-group analysis of the association of phase precession properties with age. Data are shown for EMU and AMB participants separately. **a-c,** Scatter plot of model-adjusted phase precession strength versus age. There was a significant negative association in EMU participants. AMB participants, representing a narrower age range (from 50-85 years), did not demonstrate a significant association. Combined data are shown in **c** and in Fig. 4a. **d-f**, Scatter plot of model-adjusted phase precession slope versus age. There was a significant positive association in EMU, but not AMB participants. Combined data are shown in **f** and Fig 4c.
